# Distal radial approach between theory and clinical practice.. Time to go distal!

**DOI:** 10.1186/s43044-022-00243-3

**Published:** 2022-02-05

**Authors:** Mohamed I. Sanhoury, Mohamed A. Sobhy, Mohamed A. Saddaka, Mohamed A. Nassar, Mostafa N. Elwany

**Affiliations:** Department of Cardiology and Angiology, Faculty of Medicine, Alexandria University, Alexandria, Egypt

**Keywords:** Transradial access, Radial artery, Snuffbox, Catheterization

## Abstract

**Background:**

Transradial access (TRA), which has a minimal risk of problems such as radial artery occlusion (RAO), hemorrhage, spasm, and so on, is now considered the standard procedure for cardiac catheterization. The aim of the study is to present the distal transradial access (d-TRA) as a possible promising novel technique in the field of cardiac coronary interventions comparing it to the standard conventional TRA using primary and secondary endpoints, exploring its benefits and drawbacks as a new experience in Alexandria University. One hundred cases with variable indications for coronary interventions were randomized to two arms using systematic random sampling method, coronary interventions in the first one were done via d-TRA (50 patients) and in the second arm via conventional TRA group (50 patients).

**Results:**

Technically, there were highly statistically significant differences between the two arms in favor of TRA regarding procedural success, number of punctures taken, Access time, Total procedural time, vasodilator used, and crossover to another access site; meanwhile safety profile parameters have showed statistically significant differences in favor of d-TRA regarding post-operative hematoma, AV fistula, post-operative pain and compression time, and there were no statistically significant differences regarding RAO although it occurred more in TRA group.

**Conclusions:**

In the realm of cardiac intervention, the distal radial approach is a promising technique. When compared to TRA, we found it to be a viable and safe method for coronary angiography and interventions and it could be a real option for the interventionists in the near future, with a lower risk of radial artery blockage and no significant differences in wrist hematoma and radial artery spasm. The success rate of d-TRA is proportional to the steepness of the operator's learning curve and the quality of the examples chosen.

## Background

Many developments have happened in the treatment of ischemic heart disease over the last few years, one of which is the arterial access route for both coronary angiography and percutaneous coronary procedures [1]. The transfemoral technique was replaced by the transradial approach (TRA), which has less bleeding problems, a lower hospital mortality rate, fewer access site complications, and is more cost-effective than the transfemoral approach [2]. The European Society of Cardiology issued class I recommendations in 2015 for the therapy of acute coronary syndrome, recommending the use of the TRA as the preferred route of entry for any percutaneous coronary intervention, regardless of clinical presentation [3].

Traditional radial arterial access (TRA) was initially used to perform interventional treatments in 1993. Some interventional experts were originally ecstatic about the technique's feasibility and safety [4]. TRA has been found to lower mortality in high-risk patients having urgent procedures, such as those with acute coronary syndromes. Many operators have adopted TRA as the default access for coronary operations as a result of these facts [5]. However, there is considerable skepticism about the use of TRA due to worries about the difficulty to use the radial artery (RA) as a dialysis fistula or as a conduit during coronary artery bypass grafting following TRA (CABG) [6].

To address these constraints, interventionists across the world have tried a variety of TRA modifications, including achieving patent hemostasis to prevent RA occlusion, utilizing mean artery pressure-guided compression, and obtaining TRA more distally in the anatomical snuff-box (ASB) or at the palm [7].

Recently, a novel technique called the "distal transradial approach (d-TRA) (snuffbox approach)" was developed to solve these constraints while simultaneously providing an advantage over the transfemoral approach. Kiemeneij suggested the first publication in 2017, and since then, a huge number of researches have examined the safety and practicality of this novel technique [8].

The distal radial artery (DRA), cephalic vein, and superficial branches of the radial nerve are among the contents of the anatomical snuffbox. The radial pulse can be detected in two places: the anatomic snuffbox and the first inter-metacarpal space; these two locations are alternate puncture sites for TRA [9]. By the time the radial artery reaches the anatomic snuffbox, it has already sprouted a few branches that might prevent flow stoppage in the event of a vascular blockage. The disruption of blood flow appears to be a major factor in radial artery occlusion [10].

The most prevalent problem in TRA is termed as radial artery occlusion (RAO). RAO is caused by damage to the intima of the radial artery and local blood flow stoppage, which results in thrombosis at the puncture site. However, because the d-TRA puncture site is farther away from the wrist and a smaller sheath (usually 6 Fr) is used the intimal damage in the traditional TRA site is minimal [11].

In comparison to traditional TRA, left d-TRA provides a few significant benefits. First and foremost, right-handed individuals will no longer be disturbed by their right hand's restricted mobility following catheterization [12]. Patients are instructed to place their left hand on the belly and near the right groin throughout the operations, with the thumb beneath the other four fingers, which is a natural and comfortable posture for them [13].

Furthermore, interventionists will be able to operate on the right side of the patient rather than bending over the patient, which is inconvenient. As a result, the doctor could work at a safe distance from the source of radiation [14].

## Methods

### Patients

One hundred patients were randomized into two arms using systematic random sampling method, coronary interventions in the first arm were done via d-TRA (50 patients) and in the second arm via TRA (50 patients).

### Inclusion criteria

Cases aged more than 18 years, intentional for coronary intervention either in acute or elective settings, and having palpable radial pulse at wrist and at anatomical snuff-box (ASB).

### Exclusion criteria

Those with the history of undergoing coronary intervention through radial access, history of hand surgery, history of Peripheral arterial diseases, history of carpal tunnel syndrome, history of infection or inflammation at access site, renal inadequacy (cases not on preservation hemodialysis) and history of contrast allergy or Lignocaine hypersensitivity be excluded.

### Study design

After approval of the ethics committee of the faculty of medicine, university of Alexandria with serial number 0106762, A prospective, single-center, study was done, comparing two methods of obtaining radial access in patients undergoing coronary intervention. Written informed consents were signed by the patients or their guardians.

### Randomization method

After full assessment of our study population and applying inclusion and exclusion criteria, we fully explain the goals of the study and the procedure technically to the study participants and their guardians and obtain a written formal consent from them. We choose the systematic random sampling method to divide the whole 100 cases into two groups (d-TRA and TRA), as we start using the d-TRA approach for the first case then the second one via the conventional TRA approach automatically and so on, ending with 50 patients for each group.

### Planned duration

Patients enrolled with at least 2-month follow-up in the form of assessment of the access site and clinical palpation of the radial artery pulse at both sites after the index procedure of the last patient. Direct contacts (phone numbers or E-mails) were taken from them to facilitate the communications and the follow-up.

### Assessment

Demographic data (age, sex, weight and height), risk factors history (hypertension, diabetes, dyslipidemia, smoking and family history), history of previous MI, previous PCI, previous bypass surgery, cerebrovascular disease, peripheral arterial disease, clinical indication (pre-operative angiography, chronic coronary syndrome (CCS), acute coronary syndrome (ACS), lab investigations: renal functions, Complete blood count (CBC), Coagulation profile, echocardiography to measure the left ventricular systolic function and history of drug intake of anticoagulants.

## Operative technique and steps

After defining the accessing part of the radial artery either proximally or distally, a local anesthesia is injected following proper sterilization. A 20-G cannula-needle is used for puncturing the RA, and after getting free blood backflow 0.025 inch sheath wire is introduced carefully before placement of the 5Fr or 6Fr radial sheath, followed by injection of 5 mg verapamil and 5000 IU unfractionated Heparin.

In case of difficulty of advancing the 0.025 in. sheath wire, we may use 0.14 in. wire and then replace it with the 0.025 in. sheath wire.

Adding 100 mg Nitroglycerin directly through the sheath may be considered in case of RA spasm has occurred, provided that the blood pressure measurement is maintained.

When the procedure is finished and after sheath removal a manual compression was applied for the d-TRA group to maintain hemostasis, while a radial band was used for TRA group and removed gradually over 2–3 h and radial artery pulse was assessed for each patient on both sides.

**Fig. 1:**
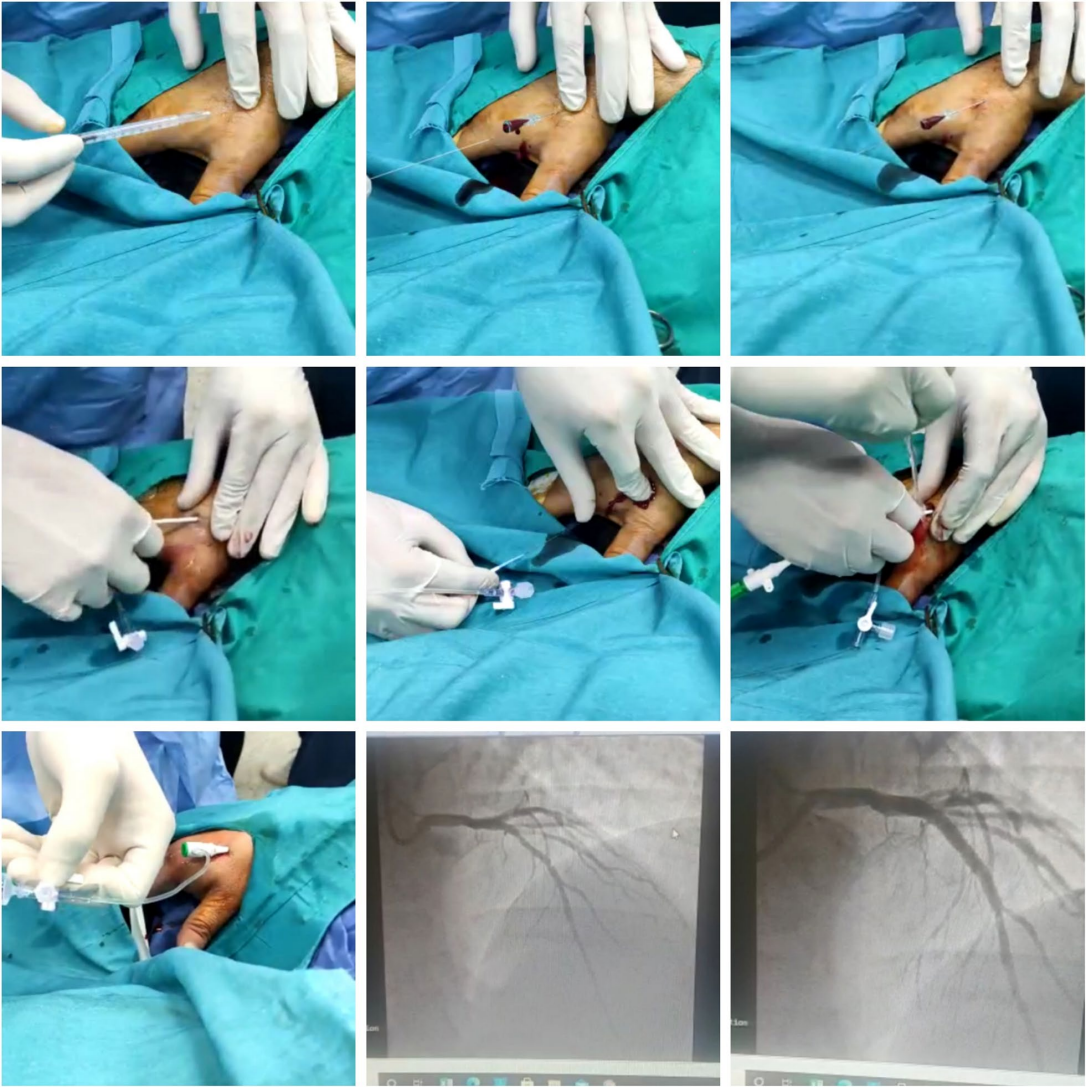
A case of anterior MI was accessed successfully via right d-TRA

**Fig. 2:**
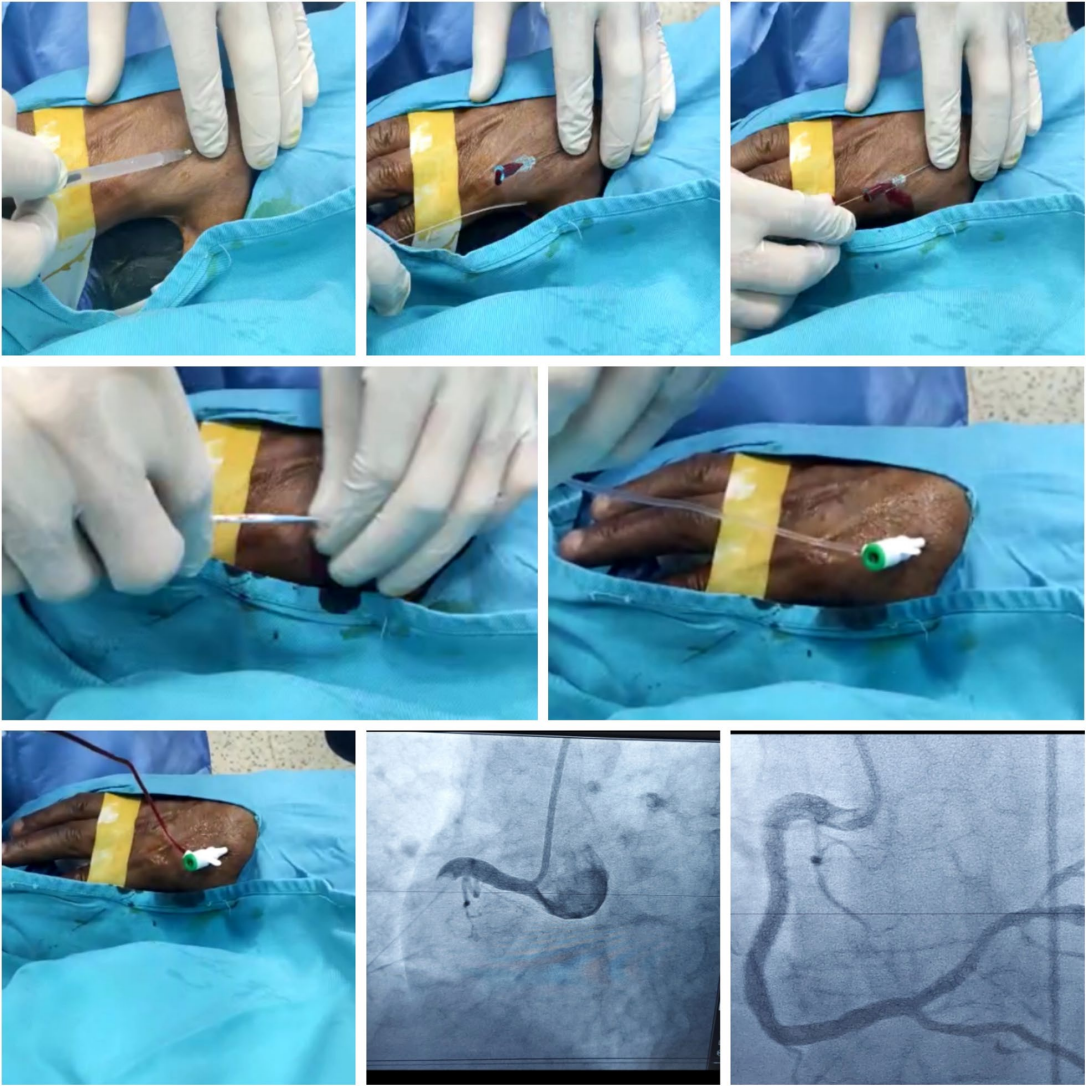
A case presented with Inferior MI was accessed successfully via right d-TRA

Major local complications or any patients' complaints during or post-procedure were noticed and recorded for each case (Figs. 1, 2).

### Primary endpoint and secondary endpoints

Procedural success, successful cannulation (sheath placement), site of the procedure (right or left), successful radial artery puncture, number of punctures used, access time (min), total procedure time (min), sheath size, frequency of vasodilator administration in sheath, quantity of catheter handed through the access site during the process, crossover to another access site, contrast volume used (ml), major complications, including Post-catheterization radial artery occlusion, hematoma, infection, arteritis, dissection, rupture of access artery and A-V fistula, post-procedure puncture site pain, post-procedure compression time and post-procedure assessment of radial artery pulse at both sites.

### Statistical analysis of the data

Data were fed to the computer and analyzed using IBM SPSS software package version 20.0***.* (**Armonk, NY: IBM Corp**)**. Qualitative data were described using number and percent. The Kolmogorov–Smirnov test was used to verify the normality of distribution Quantitative data were described using range (minimum and maximum), mean, SD, median and (IQR). Significance of the obtained results was judged at the 5% level.

## Results

Results were obtained and tabulated under two groups; group I represents patients in whom d-TRA was used and group II represents patients selected for conventional TRA.

Table 1: Sex in group I shows that 43 (86.0%) were male and 7 (14.0%) were female, while in group II 40 (80.0%) were male and 10 (20.0%) were female. There were no statistically significant differences between groups where *P* = 0.424.

**Table 1:** Comparison between the two studied groups according to demographic data

	Group I (*n* = 50)		Group II (*n* = 50)		Test of sig	*P*
	No	%	No	%		
Sex						
Male	43	86.0	40	80.0	*χ*^2^ = 0.638	0.424
Female	7	14.0	10	20.0		
Age (years)						
Min.–Max	45.0–69.0		49.0–69.0		*t* = 1.053	0.295
Mean ± SD	56.34 ± 6.08		57.56 ± 5.49			
Median (IQR)	55.0 (52.0–61.0)		57.0 (53.0–62.0)			

*IQR* interquartile range, *SD* standard deviation, *t* *Student t test*, *χ*^2^ Chi square test, *p* *p* value for comparing between the studied groups, *Group I* distal radial artery, *Group II* conventional radial artery

Age in group I was ranged between 45 and 69 years with mean ± S.D. 56.34 ± 6.08 years, while in group II was ranged between 49 and 69 years with mean ± S.D. 57.56 ± 5.49 years. There were no statistically significant differences between groups where *P* = 0.295.

Table 2: Comorbidity in group I shows that 37 (74.0%) had HTN, 25 (50.0%) had DM, 27 (54.0%) were smoker, 10 (20.0%) had dyslipidemia, 11 (22.0%) had FHX, 19 (38.0%) had history of MI, 5 (10.0%) had a history of PCI and 1 (2.0%) had a history of CABG, while in group II 43 (86.0%) had HTN, 27 (54.0%) had DM, 24 (48.0%) were smoker, 10 (20.0%) had dyslipidemia, 12 (24.0%) had FHX, 16 (32.0%) had history of MI, 8 (16.0%) had a history of PCI and 1 (2.0%) had a history of CABG. There were no statistically significant differences between groups.

**Table 2:** Comparison between the two studied groups according to comorbidity

	Group I (*n* = 50)		Group II (*n* = 50)		*χ*^2^	*p*
	No	%	No	%		
HTN	37	74.0	43	86.0	2.250	0.134
DM	25	50.0	27	54.0	0.160	0.689
Smoking	27	54.0	24	48.0	0.360	0.548
Dyslipidemia	10	20.0	10	20.0	0.000	1.000
FHX	11	22.0	12	24.0	0.056	0.812
Previous MI	19	38.0	16	32.0	0.396	0.529
Previous PCI	5	10.0	8	16.0	0.795	0.372
Previous CABG	1	2.0	1	2.0	0.000	0.000

*χ*^2^ Chi square test, *p* *p* value for comparing between the studied groups, *Group I* distal radial artery, *Group II* conventional radial artery

Table 3 shows that 21 patients used urgent setting in group I versus 27 patients in group II, while elective setting was used in 29 patients in group I versus 23 in group II with no statistically significant differences between groups. ACS, CCS and preoperative assessment show no statistically significant differences between groups. Procedural success and Cannulation success show highly statistically significant differences between groups.

**Table 3:** Comparison between the two studied groups according to different parameters

	Group I (*n* = 50)		Group II (*n* = 50)		*χ*^2^	*p*
	No	%	No	%		
Setting						
Urgent setting	21	42.0	27	54.0	1.442	0.230
Elective setting	29	58.0	23	46.0		
ACS	21	42.0	27	54.0	1.442	0.230
CCS	28	56.0	20	40.0	2.564	0.109
Pre-operative assessment	1	2.0	3	6.0	1.042	0.307
Procedural success	37	74.0	48	96.0	9.490^*^	0.002^*^
Cannulation success	37	74.0	48	96.0	9.490^*^	0.002^*^

*χ*^2^ Chi square test, *p* p value for comparing between the studied groups, *Group I* distal radial artery, *Group II* conventional radial artery

*Statistically significant at *p* ≤ 0.05

Table 4: Site in group I shows that 35 (70.0%) were in right site and 15 (30.0%) were left site, while in group II 45 (90.0%) were in right site and 5 (10.0%) were left site. There was statistically significant differences between groups where *P* = 0.012.

**Table 4:** Comparison between the two studied groups according to site and no. of punctures

	Group I (*n* = 50)		Group II (*n* = 50)		Test of sig	*P*
	No	%	No	%		
Site						
Right	35	70.0	45	90.0	*χ*^2^ = 6.250*	0.012*
Left	15	30.0	5	10.0		
No. of punctures						
Min.–Max	1.0–6.0		1.0–5.0		*U* = 762.50*	< 0.001*
Mean ± SD	2.56 ± 1.42		1.66 ± 0.89			
Median (IQR)	2.0(1.0–3.0)		1.50(1.0–2.0)			

*IQR* interquartile range, *SD* standard deviation, *U* Mann Whitney test, *χ*^2^ Chi square test, *p* p value for comparing between the studied groups, *Group I* distal radial artery, *Group II* conventional radial artery

*Statistically significant at *p* ≤ 0.05

No. of punctures in group I was ranged between 1 and 6 with mean ± S.D. 2.56 ± 1.42, while in group II was ranged between 1 and 5 with mean ± S.D. 1.66 ± 0.89. There was statistically significant differences between groups where *P* < 0.001.

Table 5: Access time in group I was ranged between 3.0 and 9.0 min with mean ± S.D. 5.10 ± 1.61 min, while in group II was ranged between 1.0 and 5.0 min with mean ± S.D. 2.28 ± 1.16 min. There was statistically significant differences between groups where *P* < 0.001.

**Table 5:** Comparison between the two studied groups according to access time, total procedural time and sheath size

	Group I (*n* = 50)	Group II (*n* = 50)	Test of sig	*p*
Access time				
Min.–Max	3.0–9.0	1.0–5.0	*U* = 170.0*	< 0.001^*^
Mean ± SD	5.10 ± 1.61	2.28 ± 1.16		
Median (IQR)	5.0 (4.0–6.0)	2.0 (1.0–3.0)		
Total procedural time				
Min.–Max	18.0–30.0	12.0–30.0	*t* = 2.530*	0.013*
Mean ± SD	24.0 ± 2.91	22.28 ± 3.83		
Median (IQR)	24.0 (22.0–26.0)	22.0 (20.0–25.0)		
Sheath size				
Min.–Max	6.0–6.0	6.0–6.0	–	–
Mean ± SD	6.0 ± 0.0	6.0 ± 0.0		
Median (IQR)	6.0 (–)	6.0 (–)		

*IQR* interquartile range, *SD* standard deviation, *U* Mann Whitney test, *t* student *t* test, *p* *p* value for comparing between the studied groups, *Group I* distal radial artery, *Group II* conventional radial artery

*Statistically significant at *p* ≤ 0.05

Total procedural time in group I was ranged between 18.0 and 30.0 min with mean ± S.D. 24.0 ± 2.91 min, while in group II was ranged between 12.0 and 30.0 min with mean ± S.D. 22.28 ± 3.83 min. There was statistically significant differences between groups where *P* = 0.013.

Sheath size in group I and group II shows that all patients in both groups had sheath size = 6Fr.

Table 6 shows highly statistically significant differences between groups according to Vasodilator used, Crossover to another access site and Post-operative puncture pain, while Radial artery occlusion, Hematoma and Av Fistula show no statistically significant differences between groups.

**Table 6:** Comparison between the two studied groups according to different parameters

	Group I (*n* = 50)		Group II (*n* = 50)		*χ*^2^	*p*
	No	%	No	%		
Vasodilator used	13	26.0	3	6.0	7.440*	0.006*
Crossover to another access site	13	26.0	2	4.0	9.490*	0.002*
Radial artery occlusion	2	4.0	7	14.0	3.053	^FE^*p* = 0.160
Hematoma	0	0.0	5	10.0	5.263	^FE^*p* = 0.056
Local infection	0	0.0	0	0.0	–	–
Arteritis	0	0.0	0	0.0	–	–
Dissection	0	0.0	0	0.0	–	–
Rupture of access site	0	0.0	0	0.0	–	–
Av Fistula	0	0.0	1	2.0	1.010	^FE^*p* = 1.000
Post-operative puncture pain	3	6.0	21	42.0	17.763*	< 0.001^*^

*χ*^2^ Chi square test, *FE* Fisher exact, *p* *p* value for comparing between the studied groups, *Group I* distal radial artery, *Group II* conventional radial artery

*Statistically significant at *p* ≤ 0.05

Table 7: No. of catheters used in group I shows that 23 (46.0%) used 2 catheters and 27 (54.0%) used 3 catheters, while in group II 27 (54.0%) used 2 catheters and 23 (46.0%) used 3 catheters. There were no statistically significant differences between groups where *P* = 0.424.

**Table 7:** Comparison between the two studied groups according to no. of catheter used and contrast volume

	Group I (*n* = 50)		Group II (*n* = 50)		Test of sig	*p*
	No	%	No	%		
No. of catheter used						
2	23	46.0	27	54.0	*χ*^2^ = 0.640	0.424
3	27	54.0	23	46.0		
Contrast volume						
Min.–Max	80.0–300.0		80.0–280.0		*t* = 0.060	0.952
Mean ± SD	189.4 ± 51.25		190.0 ± 49.16			
Median (IQR)	200.0 (150.0–230.0)		180.0 (150.0–240.0)			

*IQR* interquartile range, *SD* standard deviation, *t* student *t* test, *χ*^2^ Chi square test, *p* *p* value for comparing between the studied groups, *Group I* distal radial artery, *Group II* conventional radial artery

*Statistically significant at p ≤ 0.05

Contrast volume in group I was ranged between 80.0 and 300.0 ml with mean ± S.D. 189.4 ± 51.25 ml, while in group II was ranged between 80.0 and 280.0 ml with mean ± S.D. 190.0 ± 49.16 ml. There were no statistically significant differences between groups where *P* = 952.

Table 8: Post-operative radial artery pulse in group I shows that 45 (90.0%) had radial artery pulse felt, while in group II 44 (88.0%) had radial artery pulse felt. There were no statistically significant differences between groups where *P* = 0.749.

**Table 8:** Comparison between the two studied groups according to post-operative radial artery pulse and post-procedural compression time

	Group I (*n* = 50)		Group II (*n* = 50)		Test of sig	*p*
	No	%	No	%		
Post-operative radial artery pulse						
Negative	5	10.0	6	12.0	*χ*^2^ = 0.102	0.749
Positive	45	90.0	44	88.0		
Post-procedural compression time						
Min.–Max	2.0–7.0		19.0–40.0		*t* = 33.252*	< 0.001*
Mean ± SD	5.14 ± 0.88		24.50 ± 4.02			
Median (IQR)	5.0 (5.0–6.0)		24.0 (23.0–25.0)			

*IQR* interquartile range, *SD* standard deviation, *t* student *t* test, *χ*^2^ Chi square test, *p* *p* value for comparing between the studied groups, *Group I* distal radial artery, *Group II* conventional radial artery

*Statistically significant at *p* ≤ 0.05

Post-procedural compression time in group I was ranged between 2.0 and 7.0 min with mean ± S.D. 5.14 ± 0.88 min, while in group II was ranged between 19.0 and 40.0 min with mean ± S.D. 24.50 ± 4.02 min. There was statistically significant differences between groups where *P* < 0.001.

## Discussion

In cardiac catheterization, TRA is now considered the standard of care. Because of the low frequency of problems and the comfort provided to both the patient and the operator, (d-TRA) has gained significant interest in the field of interventional cardiology.

Because of the growing interest in this unique vascular access, numerous studies addressing the technique have been published; nevertheless, a complete and up-to-date comparison of conventional (TRA) and d-TRA is scarce [15].

Starting with the most statistically significant results in our study, access time in group I (d-TRA) varied from 3.0 to 9.0 min with a mean S.D. of 5.10 ± 1.61, whereas access time in group II (TRA) ranged from 1.0 to 5.0 min with a mean S.D. of 2.28 ± 1.16. P0.001 indicated that there were statistically significant differences between the two groups. Total procedural time varied from 18.0 to 30.0 min in group I, with a mean S.D. of 24.0 ± 2.91, and 12.0–30.0 min in group II, with a mean S.D. of 22.28 ± 3.83. *P* = 0.013 indicated that there were statistically significant differences between the groups. It's interesting to know that in d-TRA, both access time and total process time were strongly connected to the learning curve's progression. In all cases of both arms, a 6 Fr sheath was utilized.

The findings of studies comparing cannulation times were controversial. According to Kis and Soydan [16], the access time in the d-TRA group was greater than in the TRA group (46.85 ± 2.41 s against 36.66 ± 5.16 s, *p* = 0.008); these findings were consistent with those of Koutouzis et al., [17] (269 ± 251 s vs. 140 ± 161 s, *p* = 0.001). The time of cannulation was equivalent in the two techniques in the research by Hammami et al. [18], and the TRA strategy was comparable to the Turkish population [16]. Accessing the distal radial artery in the AS is more difficult than traditional TRA, and there is a learning curve to overcome. Lee et al. [19] looked at the learning curve for distal TRA and discovered that after around 150 instances, the puncture time had stabilized. Distal TRA takes substantially longer than typical TRA, according to research by Aoi et al. [20]. According to Aoi et al. [20], distal TRA took considerably longer to reach than traditional TRA (7.3 ± 5.7 vs. 5.2 ± 4.0, *p* 0.001). In the AS, the average number of attempts for distal TRA was 1.8, with ultrasonography being employed in 34.2% of patients. For traditional TRA, there was no data on attempts and ultrasonic usage.

According to our findings, Wang et al. [21], the mean puncture time for d-TRA was 5.4 ± 1.6 min and 5.6 ± 1.4 min for TRA, and the mean operational time for d-TRA and TRA was 50.0 ± 8.3 min and 51.0 ± 7.9 min, respectively. They also discovered that there were no significant differences in puncture success rate, puncture time, or operation duration between the two groups (*P* > 0.05).

According to the findings, d-TRA (group I) had 43 (86.0%) males and 7 (14.0%) females, whereas group II had 40 (80.0%) males and 10 (20.0%) females. *P* = 0.424 indicated that there were no statistically significant differences between groups. It is important to clarify that the low sample size of female sex in both groups wasn't intentional and success percentage was 100% in both groups. The age ranged from 45 to 69 years old in group I, with a mean S.D. of 56.34 ± 6.08 years, and 49–69 years old in group II, with a mean S.D. of 57.56 ± 5.49 years. There were no statistically significant differences between groups in terms of age and sex (*P* = 0.295) or success rate between males and females.

According to our findings, Rigatelli et al. [22] showed a systematic appraisal that comprised 8 eligible papers and 7693 patients (mean age 57.9 years for dTRA and 58.4 years for cTRA, respectively).

Wang et al. [21] also found that among the 160 males and 152 females in the distal radial artery puncture group, the average age was 50.17.2 years. There were 157 males and 151 females in the radial artery puncture group, with an average age of 51.27.3 years. Case number, gender, and age did not differ significantly.

In our hands, group I had 35 (70.0%) right hands and 15 (30.0%) left hands, whereas group II had 45 (90.0%) right hands and 5 (10.0%) left hands in the study. *P* = 0.012 indicated that there were statistically significant differences between groups, the selection of the side (whether right or left) of the procedure in both groups was randomized at the start of the study, but while proceeding we noticed that the left distal radial approach was more comfortable to the patient and the operator simultaneously than performing via the right side, while in group II we didn’t notice much comfort from the left side explaining the low left side radial intervention. Group I had 1–6 punctures with a mean S.D. of 2.56 ± 1.42, whereas group II had 1–5 punctures with a mean S.D. of 1.66 ± 0.89. (*P* 0.001) indicated that there were statistically significant differences between groups.

The puncture was effective in two patients from the d-TRA group, but the wire could not be pushed towards the forearm section of the radial artery; whereas the puncture failed in the rest, according to Hammami et al. [18]. For both groups, the right radial artery was the most often utilized first-intention arterial access. The left hand was approached by one patient (1%) in the TRA group and 31 patients (38%) in the d-TRA group (*p* 0.001).

Furthermore, according to Kis and Soydan [16], the left main radial artery was utilized in 12.2% (5 patients), whereas the left distal and right major radial arteries were used in 41.5% (17 patients) and 46.3% (19 patients), respectively.

Wang et al. [21] found no significant differences in puncture success rate, average puncture time, surgical time, implanted stent, or artery diameter of patients in these two groups (*P* > 0.05).

Vasodilator usage, Crossover to another access site, and post-operative puncture pain all exhibited highly statistically significant differences across groups, but radial artery blockage, hematoma, and A-V Fistula did not.

According to our findings, Wang et al. [21] found that there were no statistically significant difference (*P* > 0.05) between the comparisons for intraoperative radial artery spasm, postoperative hematoma, arterial aneurysm, and A-V fistula.

Also, according to Aoi et al. [20], post-procedural mild bleeding requiring TR band reinflation was greater in the distal TRA group (10.1% vs. 1.6%, *p* 0.001); however, hematoma was uncommon and not statistically significant (3.5% vs. 2.6%, *p* = 0.771).

No significant problems were reported in the research by Hammami et al. [18]. While significant problems occurred in 2.4% of d-TRA operations in Coomes et al. [14], the most common of which was bleeding/hematoma (18.2%). There have been a few reports of dissection and arterio-venous fistula. There were no significant differences in total problems in cohorts comparing d-TRA to TRA by Kaledin et al. [23].

Our findings revealed that in group I, 23 (46.0%) used two catheters and 27 (54.0%) used three, but in group II, 27 (54.0%) used two catheters and 23 (46.0%) used three. *P* = 0.424 indicated that there were no statistically significant differences between groups. Contrast volume was measured between 80.0 and 300.0 in group I, with a mean S.D. of 189.4 ± 51.25, and between 80.0 and 280.0 in group II, with a mean S.D. of 190.0 ± 49.16. When *P* = 952 was used, there were no statistically significant differences between the groups.

In contrast to our findings, Hammami et al. [18] found that successful catheterization was performed in 98% of TRA patients and 88% of d-TRA patients (*p* = 0.008). Despite similar doses of administered heparin between distal TRA and conventional TRA, Aoi et al. [22] found that the TR band was removed faster for d-TRA than for conventional TRA for both diagnostic catheterization (91.6 ± 31.0 vs. 126.3 ± 28.0, *p* 0.001) and PCI (120.8 ± 45.2 vs. 245.5 ± 39.5, *p* 0.001), with (pint 0.001). Furthermore, when compared to traditional TRA that had diagnostic catheterization, d-TRA that underwent PCI had a similar time to remove the TR band.

Finally, post-operative radial artery pulse was found in 45 (90.0%) of patients in group I and 44 (88.0%) of patients in group II. *P* = 0.749 indicated that there were no statistically significant differences between groups.

The post-procedural compression time in group I was 2.0–7.0 with a mean S.D. of 5.14 ± 0.88, whereas the post-procedural compression time in group II was 19.0–40.0 with a mean S.D. of 24.50 ± 4.02 *P* 0.001 indicated that there were statistically significant differences between groups. We need to clear out that manual compression post-procedure was used in group I patients, while radial band was used in group II patients denoting the major advantage of the d-TRA approach in post-procedural hemostasis.

Comparing our study results with the meta-analysis study done by Cao et al., which was published in November 2021, we both agree that there is no significant difference between d-TRA and TRA regarding post-operative hematoma (RR: 0.880, 95% CI 0.511–1.518, *p* = 0.646, I2 = 51.1%), and that there is a more incidence of RAO with TRA than d-TRA (RR: 0.203, 95% CI 0.106–0.391, *p* < 0.001, I2 = 27.1%), but we don’t share the same agreement related the access success rate as they reported that there is no much difference between both groups (RR: 0.965, 95% confidence interval (CI) 0.924–1.007, *p* = 0.1, I2 = 81.4%), which made us highlighting the value of operator experience and good selection of cases and their relation with the access success rate using d-TRA [24].

And on the other hand, comparing our work results with randomized clinical trial done by Lucreziotti S, et al., published in March 2021, we both agree on the concept of reduced access success rate with d-TRA group compared with the conventional TRA group. Vascular access failure was more frequent in d-TRA patients than in conventional TRA patients (34% versus 8.7%, *P* < 0.0001). But on contrary they didn’t report any case with RAO on both groups as we found 7 cases with conventional TRA arm. [25]

## Limitations of the study

1. The success rate of the d-TRA was highly correlated with the operator experience and learning curve especially at the start of the study.
2. The difficulty of performing a routine radial artery Doppler pre- and post-procedure to assess the variable nature of the radial artery (tortuosity, branching or calcification) and post-operative complications.

## Conclusions

In the field of cardiac intervention, the distal radial approach is a promising technique. In our study, we establish that it is a feasible and safe approach for coronary angiography and interventions, and that it is more comfortable for both the patient and the operator, especially when using the left d-TRA approach, which has a lower risk of radial artery occlusion. When compared to conventional TRA, there were no significant changes in wrist hematoma and radial artery spasm. The success rate of d-TRA is proportional to the steepness of the operator's learning curve and the quality of the examples chosen.

## Data Availability

The datasets used and/or analysed during the current study are available from the corresponding author on reasonable request.

## Abbreviations

TRA: Transradial access
d-TRA: Distal transradial access
Group 1: Distal transradial access group
Group 2: Conventional transradial access group
RAO: Radial artery occlusion
RA: Radial artery
